# Combination of diffusion tensor and functional magnetic resonance imaging during recovery from the vegetative state

**DOI:** 10.1186/1471-2377-10-77

**Published:** 2010-09-03

**Authors:** Davinia Fernández-Espejo, Carme Junque, Damian Cruse, Montserrat Bernabeu, Teresa Roig-Rovira, Neus Fábregas, Eva Rivas, Jose M Mercader

**Affiliations:** 1 Department of Psychiatry and Clinical Psychobiology, University of Barcelona, Barcelona, Spain; 2 Institute of Biomedical Research August Pi i Sunyer (IDIBAPS), Barcelona, Spain; 3 MRC Cognition and Brain Sciences Unit, Cambridge, UK; 4 Head Injury Unit, Institut Universitari de Neurorehabilitació Guttmann, Badalona, Spain; 5 Department of Neuropsychology, Institut Universitari de Neurorehabilitació Guttmann, Badalona, Spain; 6 Anesthesiology Department, Hospital Clinic, University of Barcelona, Barcelona, Spain; 7 Centre de Diagnòstic per la Imatge Hospital Clinic de Barcelona (CDIC), Hospital Clínic de Barcelona, Spain

## Abstract

**Background:**

The rate of recovery from the vegetative state (VS) is low. Currently, little is known of the mechanisms and cerebral changes that accompany those relatively rare cases of good recovery. Here, we combined functional magnetic resonance imaging (fMRI) and diffusion tensor imaging (DTI) to study the evolution of one VS patient at one month post-ictus and again twelve months later when he had recovered consciousness.

**Methods:**

fMRI was used to investigate cortical responses to passive language stimulation as well as task-induced deactivations related to the default-mode network. DTI was used to assess the integrity of the global white matter and the arcuate fasciculus. We also performed a neuropsychological assessment at the time of the second MRI examination in order to characterize the profile of cognitive deficits.

**Results:**

fMRI analysis revealed anatomically appropriate activation to speech in both the first and the second scans but a reduced pattern of task-induced deactivations in the first scan. In the second scan, following the recovery of consciousness, this pattern became more similar to that classically described for the default-mode network. DTI analysis revealed relative preservation of the arcuate fasciculus and of the global normal-appearing white matter at both time points. The neuropsychological assessment revealed recovery of receptive linguistic functioning by 12-months post-ictus.

**Conclusions:**

These results suggest that the combination of different structural and functional imaging modalities may provide a powerful means for assessing the mechanisms involved in the recovery from the VS.

## Background

The vegetative state (VS) is a disorder of consciousness (DOC) characterized by wakefulness in the absence of awareness [1]. The recovery rate from the VS is low, with only 52% of adults who are in a VS one month following a traumatic brain injury (TBI) recovering consciousness within one year [2]. Furthermore, such a recovery of consciousness is not always accompanied by a recovery of functional abilities, such as communication, or the ability to learn and perform adaptive tasks. It has been estimated that TBI patients in a VS one month after injury have a 17% chance of recovering to a level of moderate disability after 12 months, and only a 7% chance of achieving a good recovery in the same time period [3].

Little is known about the neural changes associated with recovery from the VS. Early positron emission tomography (PET) studies identified metabolic dysfunction and impaired functional connectivity in a large fronto-parietal network in a group of VS patients [4]. The recovery of consciousness of one VS patient has previously been linked to an increase in the functional connectivity within this network, [5,6] encompassing the areas known to be most active in resting-state conditions [7]. A growing body of evidence from PET and functional magnetic resonance imaging (fMRI) studies of healthy volunteers in a variety of altered states of consciousness has emphasized the role of this 'default-mode' network (DMN) in the genesis of awareness. In keeping with this, functional impairments to this network have been observed during sleepwalking, absence seizures, deep sleep and anesthesia [8-12].

fMRI has also proved its utility in identifying a number of cognitive functions which may be preserved in DOC patients, the results of which have, in some cases, proved prognostic of positive outcome [13]. In one such fMRI study investigating language processing, Coleman et al. found evidence of speech processing in three out of seven behaviorally non-communicative VS patients [14]. Six months after the scan, each of these patients had made a marked behavioral recovery relative to those patients who did not demonstrate comparable activations. Similar findings have also been reported for the neural responses observed when patients hear their own name [15].

Diffusion tensor imaging (DTI) is an emerging technique that is able to characterize brain tissue microstructure and identify changes that may not be observable with conventional MRI techniques. DTI has previously been identified as providing what may be clinically relevant biomarkers that may serve a prognostic role in the assessment of patients with TBI [16]. DTI can also serve as a tool for tracking the changes in neural tissue which accompany recovery [16]. A recent study of a group of acute TBI patients with a DOC has highlighted the potential of DTI for predicting outcome [17].

In the current study, we combined fMRI and DTI to study the evolution of a patient in a VS from one month post-TBI through to the recovery of consciousness one year later. We hypothesized that the extra information gained from the analyses of the DMN and DTI data would complement that obtained from the analysis of an fMRI language paradigm, and would therefore contribute to a greater understanding of the patient's neuropsychological profile following recovery.

## Methods

This study was approved by the Ethics Committee of the Hospital Clínic, Barcelona. Informed written consent was obtained from the patient's legal representative and from all healthy volunteers. Healthy volunteers reported no history of psychiatric or neurological disorders.

### Patient description and behavioural tests

The patient was a right-handed, 48-year-old man who suffered a severe closed head injury after falling from a ladder 33 days prior to the first MRI examination. His Glasgow Coma Scale score at the time of admission was 5 (E:1; V:1; M:3). Initial computed tomography (CT) revealed a large right parenchymal insular hematoma with subdural collection, right subarachnoid hemorrhage, intraventricular hemorrhage and a right-to-left shift, as well as subfalcial and infratentorial herniation affecting the brainstem. A right craniotomy was performed and the insular hematoma was evacuated. Post-surgical and control CT revealed an important reduction of the mass-effect without midline shift or herniation signs. Sedation was progressively withdrawn and the patient remained in a comatose state. An EEG examination showed a diffuse bilateral pattern of slow-wave activity and no response to stimuli.

At the time of the first MRI evaluation (33 days post-TBI) the patient presented with spontaneous ocular opening and flexion withdrawal in the upper extremities. The patient's score on the Disability Rating Scale [18] was 24, with a level 2 (generalised response) score on the Level of Cognitive Functioning Scale [19]. There was no evidence of visual fixation, visual pursuit, or of response to command, indicative of the VS [20]. In the months that followed, the patient made a progressive neurological recovery. At the time of discharge (three months post-ictus), he demonstrated evidence of visual pursuit and reproducible responses to simple commands, indicative of a recovery to a minimally conscious state (MCS) at this time [21].

Seven months from discharge (ten months post-ictus), the patient was re-admitted to hospital, at which point he and his relatives were approached for a re-evaluation. The patient was aware although disoriented in time. A brief bed-side neuropsychological assessment assessed the patient's language and praxis using subtests of the Spanish version of the Boston Diagnosis Aphasia Examination: conservational and expository speech, auditory comprehension, oral expression and praxis [22]. Visual neglect was assessed with Albert's test [23]. The patient showed a recovery of language comprehension and repetition skills, although his spontaneous speech and phonetic and semantic fluencies were still impaired. Although his speech was dysprosodic and dysarthric, the recovery of the patient's receptive language abilities allowed reliable communication. Normal ideomotor praxis was observed with the right hand, although bilateral neglect with a left-sided predominance, along with a major attentional impairment precluded the assessment of other complex cognitive functions.

Twelve months after the TBI, the patient was admitted to a neurorehabilitation center where he underwent a second MRI examination and a comprehensive neuropsychological assessment including the Test Barcelona-Revised, [24] Digit span and Letter-Number Sequencing (LNS) subtests of the WAIS-III, [25] Rey's Auditory Verbal Learning Test (RAVLT), [26] and a phonetic verbal fluency task [27]. Although he was orientated in person, place and time, he showed impairment in attention, working memory and visuo-perceptive functioning. All language functions (repetition, confrontation naming and comprehension tasks) were in the normal range. A second neuropsychological assessment performed two months later (14-months post-ictus) showed a significant improvement in all impaired functions including, most markedly, declarative memory (Table 1).

**Table 1:** Neuropsychological test results

Test	Raw Score 1	Raw Score 2	Maximum Score
**TBR**			
Orientation			
To person	7	7	7
To space	4	5	5
To time	23	23	23
Language			
Repetition	10	10	10
Naming	14	14	14
Comprehension	13	16	16
Visuo-perception			
Superimposed images	3	15	20

**WAIS-III**			
Attention			
Digits forwards	4	5	9
Memory			
Digits backwards	3	5	8
LNS	6	7	21

**RAVLT**			
Learning	21	35	75
Delayed recall	0	10	15
Recognition	0	11	15

**Verbal fluency**			
/p/	n.a.	25	

TBR: Test Barcelona-Revised; WAIS-III: Wechsler Adult Intelligence Scale III; LNS: Letter-number sequencing; RAVLT: Rey Auditory-Verbal Learning Test; n.a.: not assessed (due to severe dysarthria).

### MRI acquisition

MRI-data were acquired in a 3 T scanner (Magnetom Trio Tim, Siemens, Germany) at the Center for Image Diagnosis of the Hospital Clinic, Barcelona. fMRI was acquired in a single experimental session using a gradient-echo echo-planar imaging (EPI) sequence including 240 volumes (TR = 2000 ms, TE = 29 ms, 36 slices, matrix size = 128 × 128, slice thickness = 3 mm). For anatomical reference, a high-resolution (1 × 1 × 1 mm) T1-weighted magnetization-prepared rapid gradient-echo dataset was acquired (TR = 2300 ms, TE = 2.98 ms, matrix size = 256 × 256). Diffusion-weighted images were sensitized in 30 non-collinear directions with a b-value = 1000 s/mm^2^, using an EPI sequence (TR = 5600 ms, TE = 89 ms, 49 slices; slice thickness = 1 mm, gap = 0.6 mm, matrix size = 122 × 122). Total scanning time was approximately 30 minutes per subject.

In order to compare DTI images obtained from the patient with healthy controls, we also acquired DTI-datasets using the same sequence from nineteen, right-handed, neurologically-normal subjects (8 male, 11 female) aged between 19-49 years. Right-handedness was assessed with the Edinburgh Handedness Inventory [28].

All collected data underwent quality control tests prior to the analysis. D.F.E visually inspected the raw images for the presence of motion related artifacts in all cases. No volumes were discarded as a result of this process.

### fMRI paradigm

The stimuli used are described in detail in a previous study [29]. In brief, these consisted of eight, twenty-second long spoken narratives regarding everyday events. Participants heard these narratives played both normally (forward narratives) and reversed (backward narratives). Reversed narratives match the originals in terms of acoustic characteristics but violate several properties of human speech, thus allowing the isolation of those processes specifically related to the processing of language relative to those associated with the processing of simple sounds [30,31]. Eight blocks of a baseline silence condition of the same duration were used to separate the narratives. Stimuli were presented using 'Presentation' (v.10.1, Neurobehavioural System), running on a Windows XP PC with an MRI-compatible high-quality digital sound system incorporating noise-attenuated headphones (VisuaStim Digital. Resonance Technology, Inc.).

### fMRI-data analysis

The fMRI data were pre-processed and analyzed using SPM5 http://www.fil.ion.ucl.ac.uk/spm running in Matlab 7.0 (MathWorks, MA).

For the task-related activation and deactivation analysis, fMRI images were first realigned to the first image in the dataset in order to reduce movement artifacts. A rigid-body transformation was applied to co-register structural and functional data. Spatial normalization parameters were calculated from the structural image and applied to the co-registered functional data (re-sampled voxel size of 3 mm) [32]. Normalized images were smoothed with an 8 mm Gaussian kernel. The analysis was based on the general linear model using the canonical hemodynamic response function [33]. Each scan was modeled to belong to the speech, non-speech or silence condition. Motion was below 1.5 mm of translation and 2 degrees of rotation. Nevertheless, to control any subtle effect on the statistical analysis, parameters calculated from the realignment step were also included as covariates of no interest. High-pass filtering using a cut-off period of 128 seconds was implemented in order to remove slow-signal drifts from the time series.

Low-level auditory processing was assessed in a comparison of the fMRI responses to both auditory conditions with those associated with the silence condition. Speech processing was assessed by a comparison of the fMRI responses to forward narratives with those to backward narratives. Task-induced deactivations were assessed by the contrast between silence and forward narratives. The analysis of task-related deactivations allows the identification of regions considered to be associated with the DMN [34]. The statistical threshold was set at p < 0.05 False Discovery Rate (FDR) corrected [35]. Only clusters containing more than 10 contiguous voxels were considered. When no significant activations were found at this level we reduced the statistical threshold to p < 0.001-uncorrected to exclude the possibility of failing to detect more subtle changes in the BOLD signal due to this conservative approach. To prevent the false positive risk, and taking into account the strong a priori hypothesis alongside the fact that a whole-brain approach might be a rather conservative analysis considering the specificity of the cognitive function we sought to assess, a post-hoc analysis was applied using small volume correction to reduce the number of comparisons to specific regions. 15 mm radius spheres centered on the most significant foci reported in a meta-analysis of speech processing task (MNI-coordinates: -57,-40,2) [36] and the three most significant foci reported in a meta-analysis of task-induced deactivations: i.e. BA 31/7 (MNI-coordinates: -5,-49,40), BA39 (MNI-coordinates: -45,-67,36) and BA 10 (MNI-coordinates: -1,47,-4) were used for the 'forward > backward narratives' and 'silence > forward narratives' contrasts respectively [37]. A sphere of this size was chosen in order to effectively cover any region of interest as well as provide sufficient sensitivity for small volume correction purposes.

A 'resting state' functional connectivity analysis was also performed in order to further characterize the DMN in this patient. Following the method proposed by Fair *et al. *[38] we analyzed the interleaved resting blocks from our fMRI task. These authors demonstrated that such a method is well-suited for resting state functional connectivity analysis leading to similar results to those obtained from resting state acquisitions. In order to minimize the effect of the task blocks on each resting state epoch, 7 volumes (14 seconds) were excluded from the beginning of every resting state block (at the end of each task block) and 3 volumes (6 seconds) were included after the start of each task block, to allow for the hemodynamic response to return to baseline and to account for the hemodynamic delay respectively. This procedure provided 90 seconds of resting state data for each session. Pre-processing steps included realignment, spatial normalization into MNI space and spatial smoothing using a Gaussian kernel of 8 mm. Then we applied a temporal band-pass filter (0.009 Hz <f< 0.08 Hz) using Resting-State fMRI Data Analysis Tookit v1.4 http://www.restfmri.net/forum/REST_V1.4. Time course of voxels in a seed region of interest located in the posterior cingulate cortex (PCC) (6-mm sphere centered on coordinates -5,-49,40) [37,39] was extracted using MarsBar [40]. Similar time course extractions were performed for a white matter and a ventricular sphere ROIs as well as for a whole brain mask. Those, along with the six parameters obtained by rigid body head motion correction, were included as regressors of non interest in the statistical model. Correlation maps were produced by computing the correlation coefficient between the time course extracted from the PCC ROI and all voxels included in the three spherical ROIs used for small volume correction in the equivalent deactivation analysis (see above). Results were thresholded at p < 0.05 FDR-corrected.

### DTI-data analysis

Images were processed using the FMRIB Software Library (FSL, v4.1.0; http://www.fmrib.ox.ac.uk/fsl). Pre-processing steps included eddy-current correction and skull and non-brain tissue removal [41,42]. Fractional anisotropy (FA) and mean diffusivity (MD) maps were obtained using FDT. T1-weighted data were skull-stripped and segmented to obtain white matter binary masks [43]. After segmentation, T1-weighted data and white matter masks were registered with the T2-weighted (b0) images using a mutual-information cost function and 12 degrees of freedom [44]. After registration, masks were visually inspected and misclassified voxels were removed using FSLView masking tools. The large right parieto-temporal lesion was manually outlined on the T2-weighted images and removed from the WM masks obtaining normal-appearing white matter (NAWM) masks.

An in-house script running on Matlab 7.0 was used to generate histograms from the FA and MD maps. This comprised 250 bins, which were normalized by the total number of voxels contributing to the histogram in order to compensate for brain size differences. Histograms were characterized by mean, peak position and peak height. An arcuate fasciculus mask, defined as the temporal projection of the superior longitudinal fasciculus, was obtained from the JHU white-matter tractography atlas [45]. It was warped into native space using FLIRT [44]. Affine-registration (12 degrees of freedom) and mutual-information cost function were applied to register the T2-weighted image from each subject to the MNI template of the FSL (T1-weighted). The inverse of the transformation matrix was applied to the mask in standard space to un-warp it into native space for each subject. For the patient, we registered the second T2-weighted image to the first one using rigid-body transformation (6 degrees of freedom) and normalized correlation function, and used the mask warped to the first one for both sessions. To ensure that only white matter fibers were included, we applied a threshold FA value of 0.2 [45]. Mean FA and MD values were obtained from the thresholded regions of interest masks in native space for each subject.

## Results

At the time of the initial MRI (1-month post-ictus), the whole-brain analysis showed no significant activation for the contrast 'forward narratives > backward narratives' at a FDR-correction level. However, when we reduced the statistical threshold to p < 0.001-uncorrected we found anatomically appropriate activation comprising a single cluster in the left superior and middle temporal gyrus. To prevent the false positive risk, we applied a small volume correction to reduce the number of comparisons. Post-hoc analysis using small volume correction provided significant results after correction for multiple comparisons (FDR corrected p < 0.05). Data from the fMRI task have been reported elsewhere previously [29] and were shown to demonstrate anatomically appropriate speech recognition activations using a more sensitive region of interest procedure. In that same study, [29] a group of healthy volunteers showed comparable activation in the left superior and middle temporal gyrus (Brodmann areas [BA] [21,22]) and, less significantly, in the left inferior frontal gyrus in this contrast.

**Figure 1:**
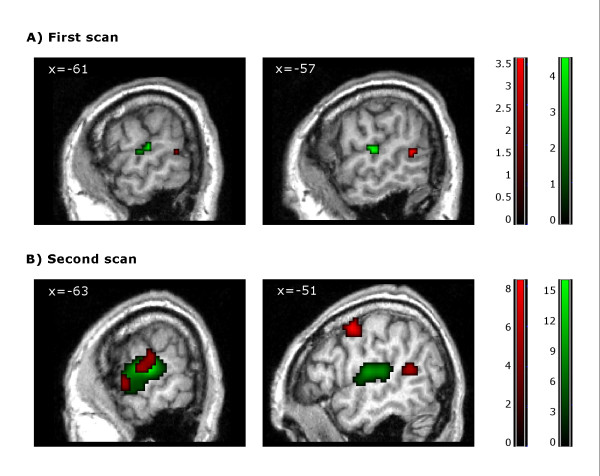
**Task -related activations**. Patient's brain activation in the first (A) and second (B) scans for the contrast 'forward > backward narratives' (red) and the contrast 'narratives > silence' (green). Results are thresholded at p < 0.05 FDR-corrected and mapped on the patient's brain. Notice that the cluster displayed in the first scan for the contrast 'forward > backward narratives' is thresholded at p < 0.05 FDR-small volume correction. Color-bars indicate *t *statistic values. Numbers on the left superior corner of each image refer to the MNI-coordinate of the peak maxima in the *x *axis for each contrast.

**Figure 2:**
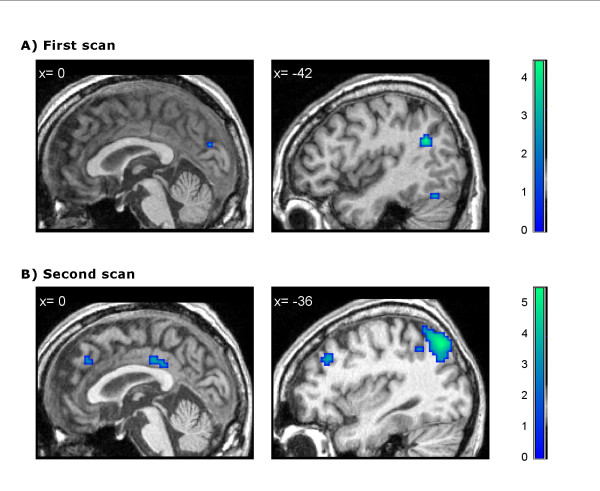
**Task- related deactivations**. Patient's task-induced deactivations (contrast 'silence > forward narratives') in the first (A) and second (B) scans (medial and lateral views). Results are thresholded at p < 0.001 uncorrected and mapped on the patient's brain. Color-bars indicate t statistic values. Numbers on the upper-left corner of each image refer to MNI-coordinate in the *x *axis. The images on the right correspond to the peak-maxima of the main cluster for each contrast.

The 'narratives > silence' contrast revealed two significant clusters comprising the left superior and transverse temporal gyrus and the right parahippocampal gyrus respectively (Table 2, Figure 1). The 'silence > forward narratives' contrast failed to reveal any significant deactivations at FDR-corrected level. However, when the statistical threshold was reduced to an uncorrected p < 0.001 it revealed significant deactivations in some of the areas related to the DMN such as the left BA 39 or the precuneus, as well as other more unexpected areas: cuneus, fusiform gyrus, thalamus and lingual gyrus. Post-hoc analysis using small volume correction confirmed significant deactivation only in BA39 (Table 2, Figure 2).

**Table 2:** Patient's cerebral activations and de-activations during the fMRI task

Contrast	Brain structure	BA	k	Side	Coordinates			z-value	p-FDR
							
					x	y	z		
**1 month of evolution (vegetative state)**									

Forward > Backward narratives*	Superior temporal gyrus, Middle temporal gyrus	21, 22	17	L	-57	-51	9	3.57	0.046

Narratives > Silence	Trasverse temporal gyrus, Superior temporal gyrus	41,42	13	L	-57	-18	9	4.81	0.017
	Parahipocampal gyrus	19	14	R	39	-42	-6	4.73	0.017

Silence > Forward narratives*	Superior Temporal Gyrus,	39	16	L	-42	-57	27	4.33	0.003

**13 months of evolution (conscious)**									

Forward > Backward narratives	Precentral gyrus	6	42	L	-51	0	48	7.77	< .001
	Superior temporal gyrus, middle temporal gyrus	21, 22	54	L	-51	-51	12	5.61	< .001
	Superior temporal gyrus	22	15	L	-63	-3	-3	5.19	< .001
	Trasverse temporal gyrus, Postcentral gyrus	40, 42	33	L	-66	-18	12	5.04	< .001

Narratives > Silence	Superior temporal gyrus	41,42	461	L	-63	-24	9	Inf.	< .001
	Paracentral lobule	4	24	L	-6	-39	75	7	< .001

Silence > Forward narratives	Inferior parietal lobule	7, 40	234	L	-36	-66	48	5.35	0.001
	Precuneus	7	17	L	-15	-63	60	4.33	0.005
	Superior frontal gyrus	6	17	L	-18	30	60	4.12	0.01

The last four columns (coordinates and z-value) refer to the main peak of activation in the cluster. Coordinates refer to Montreal Neurological Institute. Results thresholded at p < 0.05 FDR-corrected. BA = Brodmann areas; k = cluster size; L = left hemisphere, R = right hemisphere. * = significant at p < 0.05 FDR small volume correction.

The analysis of the second fMRI acquisition (12-months post-ictus, when the patient had regained consciousness) revealed four significant clusters in cortical language regions for the contrast 'forward > backward narratives' (FDR-corrected p < 0.05): left superior and middle temporal gyrus, left precentral gyrus, left postcentral gyrus and transverse temporal gyrus. The contrast 'narratives > silence' revealed two significant clusters comprising the left superior temporal gyrus and paracentral lobule respectively (FDR-corrected p < 0.05). Finally, the contrast 'silence > forward narratives' revealed significant deactivations (FDR-corrected p < 0.05) in the inferior parietal lobule, left precuneus and left superior frontal gyrus (Table 2, Figures 1, 2).

**Figure 3:**
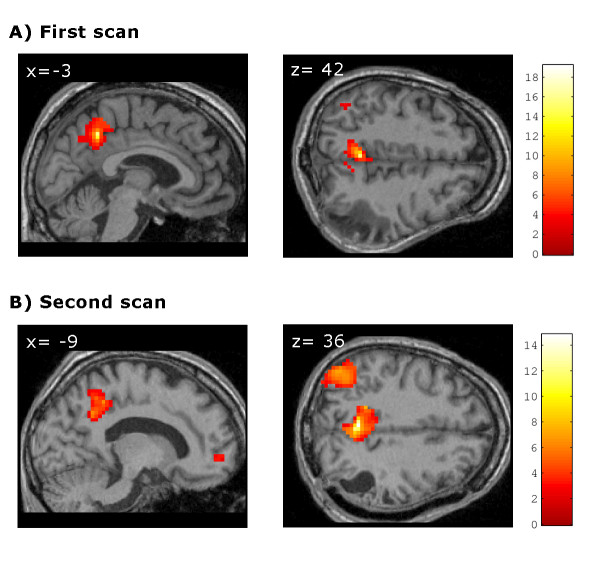
**Correlations with posterior cingulate/precuneus BOLD activity**. Patient's positive correlation with posterior cingulate/precuneus BOLD activity in the first (A) and second (B) scans. Results are thresholded at p < 0.05 FDR-corrected and mapped on the patient's brain. Numbers on the upper-left corner of each image refer to MNI-coordinate.

The functional connectivity analysis revealed that in the first MRI scanning session the patient showed significant correlations between PCC and two small clusters located in the parieto-temporal junction whereas in the second session he exhibited a more significant correlation pattern between the PCC and both the parieto-temporal junction and the medial prefrontal cortex (Table 3, Figure 3).

**Table 3:** Patient's positive correlations with posterior cingulate/precuneus BOLD activity

Brain structure	BA	k	Side	Coordinates			z-value	p-FDR
						
				x	y	z		
**1 month of evolution (vegetative state)**								

Precuneus	31,7	235	L	-6	-51	39	Inf.	< .001
TPJ	39,40	21	L	-42	-63	24	4.13	< .001
TPJ	40	10	L	-45	-60	45	3.25	0.001

**13 months of evolution (conscious)**								

PCC/Precuneus	31	338	L	-3	-51	36	5.66	< .001
TPJ	39,40	386	L	-42	-72	30	4.49	< 0.001
MPF	10	19	L	-15	54	-3	2.98	0.001

The last four columns (coordinates and z-value) refer to the main peak of activation in the cluster. Coordinates refer to Montreal Neurological Institute. Results thresholded at p < 0.05 FDR-corrected. PCC = posterior cingulate cortex; TPJ = temporo-parietal junction; MPF = medial prefrontal cortex; BA = Brodmann areas; k = cluster size; L = left hemisphere, R = right hemisphere.

**Figure 4:**
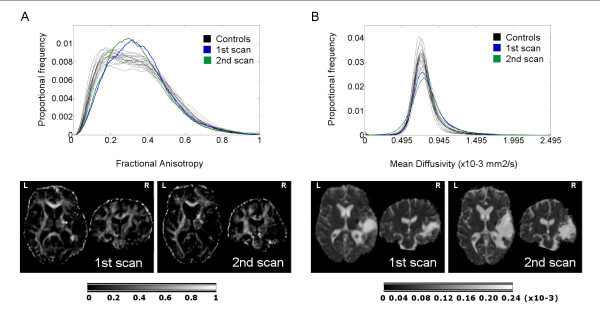
**Histograms of normal-appearing white matter**. Fractional anisotropy (A) and mean diffusivity (B) histograms of normal-appearing white matter of the patient in the first (blue line) and second (green line) scans and the 19 normal control subjects (black lines) over all voxels in the brain (top of the panel). The bottoms of each panel display the correspondent fractional anisotropy and mean diffusivity maps. Color-bars indicate fractional anisotropy (arbitrary units) and mean diffusivity (mm^2^/sec) values.

In both sessions the mean, peak position, and peak height from the NAWM FA histograms of the patient were within the normal range. The patient's MD histogram means were within the normal range in both scans while peak positions were within the normal range only in the first scan and increased in the second one. Peak height was reduced relative to the normal range in both the first and the second scan. Mean FA values from the arcuate fasciculus were also slightly reduced, while MD mean values were within the normal range in both scans (Table 4, Figure 4).

**Table 4:** FA and MD parameters for NAMW and arcuate fasciculus

			**Patient 1**^**st **^**scan**	**Patient 2**^**nd **^**scan**	Controls range
NAWM	FA	Mean	3.5 × 10^-1^	3.4 × 10^-1^	3.1-3.6 × 10^-1^
		Peak position	3.3 × 10^-1^	2.9 × 10^-1^	1.3-3.6 × 10^-1^
		Peak height	1.0 × 10^-2^	1.0 × 10^-2^	1.0-1.1 × 10^-2^
	
	MD	Mean	8.5 × 10^-4^	8.4 × 10^-4^	7.7-8.6 × 10^-4^
		Peak position	7.6 × 10^-4^	8.2 × 10^-4^	7.2-7.9 × 10^-4^
		Peak height	2.7 × 10^-2^	2.5 × 10^-2^	2.9-4.3 × 10^-2^

Arcuate fascicle	FA	Mean	3.6 × 10^-1^	3.7 × 10^-1^	3.8-4.3 × 10^-1^
	
	MD	Mean	8.1 × 10^-4^	8.0 × 10^-4^	7.0-8.1 × 10^-4^

NAWM = normal-appearing white matter; FA = fractional anisotropy values; MD = mean diffusivity values (mm^2^/s).

## Discussion

The current study contrasted the fMRI and DTI scans of a TBI-patient at one-month post-ictus, when he was in a VS, with those made one-year later when the patient had recovered consciousness. The fMRI data showed a pattern of cerebral activation in brain regions associated with language processing in both the first scan, when he was unable to make an overt response to these stimuli, and in the second scan, when he was able to communicate verbally. At one-month post-ictus, this activation was restricted to a single locus in the posterior superior-middle temporal gyrus (Wernicke's area) while in the second scan, 12-months later, it also comprised more regions within these two gyri as well as, interestingly, areas of higher associative order, such as Brodmann area 40. This heteromodal area is known to be involved in the association of visual and auditory linguistic information, and so activation may reflect a recovery of integrative functioning [46,47].

The presence of anatomically appropriate activations in response to speech in VS patients has been linked with a positive outcome [14,15,48,49]. However, other cases have been reported in which the presence of these responses were not associated with recovery [50,51]. A recent review of eight PET studies and six fMRI studies including a total of 48 VS patients estimated that the activation of high level associative cortical regions can predict eventual recovery of consciousness with a specificity of 93% and a sensitivity of 69% [52]. The presence of similar fMRI markers in the patient reported in the current study is consistent with this trend. It is clear, however, that a greater understanding of the prognostic value of appropriate cerebral responses to language in this population can be gained from future longitudinal studies of large groups of these patients. This notwithstanding, the extent to which the linguistic functioning of the current patient improved upon his return to consciousness, points to the potential for the findings of an initial early scan to act as a predictor of future linguistic function.

In the first scan, the patient also showed a reduced pattern of deactivations relative to the second scan that were restricted to the left parieto-temporal junction. When the patient had regained consciousness, this pattern became more similar to the pattern classically reported in healthy volunteers as being part of the DMN, engaging parietal and frontal areas in the non-damaged hemisphere. Functional connectivity analysis confirmed and complemented these results as the patient showed absent parieto - frontal functional connectivity in the first scan and restored functional connectivity within these regions in the second. These findings are in agreement with early metabolic studies with VS patients [6] and more recent work with healthy subjects which have suggested that the activity in this fronto-parietal network may be related to conscious perception [53]. Furthermore, recently it has been demonstrated that functional connectivity within the DMN may be partially preserved in some VS patients, though significantly reduced compared to healthy volunteers [39]. This reduction has also been related to the level of consciousness experienced in states of altered consciousness [54]. The authors of these works have proposed two different levels of functional connectivity to explain their findings: one that would persist independently of the level of consciousness, and another that is more related to the presence of conscious cognitive processes. The presence of deactivations within parietal areas only in the initial scan, along with the outcomes of the functional connectivity analysis showing connectivity restricted to these regions, may suggest that they are involved in the lower level processes they describe, while the more frontal areas observed in the second scan may be more related to the higher conscious processes. This proposal, however, is necessarily speculative.

Unlike the findings of the studies reported above, our deactivation results cannot be interpreted as functional disconnections, and our functional connectivity findings should be interpreted with caution as the resting state data obtained after removal of interleaved task blocks from our fMRI acquisition is significantly shorter than those used in previous resting state studies. We were unable to acquire resting state data for a more robust functional connectivity analysis due to the time demands of the other scanning protocols on the patient. However, deactivations in the DMN areas have been shown to be largely task independent, varying little in their locations across a wide range of tasks [7]. A high level of consistency in the anatomy of the DMN has been demonstrated across these methodological approaches (i.e. resting state functional connectivity studies and task-induced deactivations in classical fMRI paradigms as reported here), [34,55] consistent with the results reported here. It seems reasonable to assume, then, that the dysfunctional pattern of deactivations exhibited by the patient in the first scan are a reflection of some level of structural or functional disorganization associated with a post-acute state. A recent report of a post-traumatic VS patient who recovered some degree of consciousness following the stimulation of the dorsolateral prefrontal cortex has stressed the importance of the maintenance of the fronto-parietal network in the recovery of consciousness [56]. The current findings of a greater fronto-parietal network of deactivations observed when consciousness had been regained relative to those observed when the patient was in the VS, are consistent with this notion. Future studies of this sort, however, may benefit from the information gained from a more robust functional connectivity analysis obtained from a resting state acquisition, over and above that obtained from the analysis of deactivations alone.

The DTI data support and complement those results obtained from the fMRI. The relative structural integrity of the arcuate fasciculus observed when the patient was in the VS reinforces the predictions that can be made on the basis of the preserved fMRI language activations. The global analysis of NAWM revealed an absence of any major impairment in the WM tissue, without any outstanding changes between the first and second scans. One of the most commonly reported consequences of TBI observed in post-mortem studies of TBI patients who remained in a VS until death, is grade II/III diffuse axonal injury (DAI) [57-60]. However, the mechanisms commonly involved in DAI, i.e. stretching and shearing of white matter fibers due to rotational forces normally related to acceleration-deceleration effects,[61] did not come into play in the current case, as the cause of the TBI was a fall. The accident caused an extensive concussive lesion restricted to the right parieto-temporal area but it did not seem to affect areas far away from the lesion boundaries. DAI can disrupt critical cortical-subcortical pathways, leading to severe cognitive dysfunction and precluding an effective reorganization of the preserved structures [62]. The absence of structural evidence of DAI in our patient revealed by the DTI analysis may highlight the potential for this as a measure of the extent to which recovery made on the basis of functional re-organization can take place, once the large hemorrhage and post-evacuation complications have subsided.

As discussed earlier, the current results are taken from a single VS patient, and therefore must be interpreted with care in relation to the wider VS population. Currently, there are three other published longitudinal neuroimaging studies of recovery from DOC, following two VS patients [6,63] and one MCS patient, [64] which have provided insights into the mechanisms involved is such recovery. These studies, however, focused on either functional or structural data, or contrasted a number of scans performed after recovery. The current study, therefore, is the first time that data from fMRI, DTI and neuropsychological assessment have been combined in the study of the cerebral and clinical changes of a VS patient from the time of VS through to recovery. The low incidence of a good recovery in VS patients has therefore made this case an exceptional opportunity in which to study the mechanisms associated with this process.

## Conclusions

The current findings provide evidence that structural and functional preservation of linguistic cerebral networks may have prognostic value for the language abilities of VS patients following the recovery of consciousness. In addition, dysfunction in areas related to the DMN may have a role in explaining disorders of consciousness. In the absence of significant structural damage to long-range connections, the functional recovery of this network may accompany the restoration of consciousness.

Taken together, our findings suggest that a multi-modal imaging approach can provide a powerful tool for assessing the mechanisms involved in the recovery of consciousness in DOC patients. Further longitudinal studies with large cohorts will prove useful in assessing its full value in predicting outcome. Such insights may then provide guidance for decisions relating to rehabilitation programs by orientating these towards the effective stimulation of those cognitive functions that appear preserved, in order to maintain their functional and structural integrity.

## Competing interests

The authors declare that they have no competing interests.

## Authors' contributions

DFE and CJ made substantial contribution to conception and design, interpretation of data as well as in the preparation of the first draft and further revisions of the manuscript. Neuroimaging data were collected and analyzed by DFE. DC contributed heavily to the final revision, providing comments and perspectives. MB and TR participated in the collection of neuropsychological data. ER and NF were involved in the collection of acute clinical data. MB, TR, ER, NF and JMM made a critical revision of the manuscript for important intellectual content providing additional comments and contributions. CJ supervised the study. All authors read and approved the final manuscript.

## Pre-publication history

The pre-publication history for this paper can be accessed here:

http://www.biomedcentral.com/1471-2377/10/77/prepub

## Aknowledgements

This work was supported by grants SAF2007-66077 from the Spanish Ministry of Science and Innovation and 2009SGR0941 from the Generalitat de Catalunya to the Neuropsychology Research Group. D. Fernández-Espejo was supported by a fellowship from the Spanish Ministry for Education (AP2006-00862). D. Cruse was supported by a Medical Research Council Translational Grant (U.1055.01.002.00007.01).
